# Ethics of Medicine: An Anniversary Address Delivered the 3d of April, 1844, before the Medico-Chirurgical Society of Louisiana

**Published:** 1845-01

**Authors:** 


Art. V.—*Ethics of Medicine: An Anniversary Address delivered the* 3*d of April*, 1844, *before the Medico-Chirurgical Society of Louisiana.* By Thomas M. Logan, M.D. Published by the Society. New Orleans, 1844.

